# COVID-19 lockdown: Impairments of objective measurements of selected physical activity, cardiorespiratory and sleep parameters in trained fitness coaches

**DOI:** 10.17179/excli2022-4986

**Published:** 2022-08-16

**Authors:** Ismail Dergaa, Achraf Ammar, Amine Souissi, Mohamed Saifeddin Fessi, Khaled Trabelsi, Jordan M. Glenn, Amine Ghram, Morteza Taheri, Khadijeh Irandoust, Hamdi Chtourou, Mohamed Romdhani, Helmi Ben Saad, Karim Chamari

**Affiliations:** 1 Primary Health Care Corporation (PHCC), Doha P.O. Box 26555, Qatar; 2 Research Unit Physical Activity, Sport, and Health, UR18JS01, National Observatory of Sport, Tunis 1003, Tunisia; 3 Institute of Sport Sciences, Otto-von-Guericke University, 39104 Magdeburg, Germany; 4 Interdisciplinary Laboratory in Neurosciences, Physiology and Psychology: Physical Activity, Health and Learning (LINP2), UFR STAPS, UPL, Paris Nanterre University, Nanterre, France; 5 Research Laboratory Education, Motricité, Sport et Santé, EM2S, LR19JS01, High Institute of Sport and Physical Education of Sfax, University of Sfax, Sfax, Tunisia; 6 Exercise Science Research Center, Department of Health, Human Performance and Recreation, University of Arkansas, Fayetteville, AR 72701, USA; 7 Healthy Living for Pandemic Event Protection (HL - PIVOT) Network, Chicago, IL, USA; 8 Department of Exercise Physiology, Faculty of Physical Education and Sport Sciences, University of Tehran, Tehran, Iran; 9 Department of Sport Sciences, Imam Khomeini International University, Qazvin; 10 Université de Sousse, Hôpital Farhat HACHED, Laboratoire de Recherche "Insuffisance cardiaque" (LR12SP09), Sousse, Tunisie; 11 Aspetar, Orthopaedic and Sports Medicine Hospital, FIFA Medical Centre of Excellence, Doha P.O. Box 29222, Qatar; 12 ISSEP Ksar-Said, Manouba University, Tunisia

**Keywords:** heart pulse, home confinement, sedentary behavior, sleep characteristics, step count

## Abstract

The COVID-19 outbreak resulted in the shutdown of athletic training facilities. Although the effects of these restrictions on daily activity and sleep patterns have been widely analyzed, the employed tools often lacked accuracy, and were based on subjective measures. This study assessed the effects of home confinement on objective physical activity (PA), physiological and sleep parameters in active individuals. Sixteen male elite fitness coaches (age: 29±3 years; height: 183±6 cm; body mass: 82±5 kg, body mass index: 24.7±1.8 kg/m^2^) participated in this retrospective study. One-way analysis of variance was conducted to analyze selected PA, physiological and sleep parameters collected by smartwatch (Garmin Fenix 6 pro, USA) data during four consecutive months [*i.e*., pre-confinement, 1^st^ and 2^nd^ months of confinement, and post-confinement, year 2020]. Ramadan intermittent fasting (RIF) month occurred during the 2^nd^ month of confinement. Compared to pre-confinement, significant changes were registered for almost all parameters during the 1^st^ and/or the 2^nd^ month of confinements (p<0.001), with ***(i)*** higher values for resting heart rate, sleep latency, and total, light and rapid eye movements sleep times (% change=7-523 %), and ***(ii)*** lower values for PA parameters, calories/day spent, average and highest respiratory rates, and deep sleep time during the home confinement period (% change=5-36 %). During the post-confinement month, all parameters regained pre-confinement values. In conclusion, home confinement-induced detraining negatively influenced the objective measurements of cardiorespiratory and sleep parameters among fitness coaches with a deeper effect during the 2^nd^ month of home confinement, possibly due to the effect of RIF.

## Introduction

The severe acute respiratory syndrome coronavirus 2 (SARS-CoV-2) is responsible of the coronavirus disease (COVID-19) (Erickson et al., 2011[19]); with 118,000 cases and 4,000 deaths globally, the World Health Organisation (WHO) declared a global pandemic on 11 March 2020. Unfortunately, by June 23^rd^ 2022, over 547 million confirmed COVID-19 cases and >6.34 million deaths were reported worldwide. Considering the challenges imposed by the COVID-19 pandemic to the global health care system and society in general (Dergaa et al., 2022[17][16]), and in a hope to cut the rate of new infections and flatten the COVID-19 contagion curve, the majority of countries worldwide imposed mass home-confinement directives, with most including quarantine and physical distancing (Musa et al., 2021[29], 2022[30][31]; Chtourou et al., 2020[12]; Dergaa et al., 2021[18]). Home confinement, and the resulting social isolation (Ammar et al., 2020[5]), are major stressors that contribute to widespread emotional distress (Ammar et al., 2021[3]), and may aggravate pre-existing conditions (Russ et al., 2012[38]; Musa et al., 2022[30][31]), thus causing issues such as sleep disorders and/or weakened immune systems, among others (Cohen et al., 2012[13]; Musa et al., 2022[31]). Additionally, active people had to face an unprecedented interruption to their normal routines, along with substantial attenuations in their daily physical activities, resulting in detrimental morphological and physiological adaptations (Trabelsi et al., 2022[47]). However, given the strict government rules and the difficulty of planning scientific investigations and recruiting participants in lockdown conditions (Romdhani et al., 2022[37]; Ammar et al., 2020[4][5]; Akbari et al., 2021[1]), collecting objective data (i.e. not self-report) on how physical activity (PA), physiological and sleep parameters changed from pre-, during-, and post-home confinement in physically active individuals was difficult. Most studies conducted during these global lockdowns mainly employed online surveys. In this context, research has shown that limited or precluded access to equipment and sports facilities (*e.g.*, coaching staff) compromised athlete's daily routine of the “train, sleep and eat,” while another study reported that COVID-19 lockdown negatively impacted sleep patterns in South African elite and semi-elite athletes. Recently, Wingerson et al. (2021[49]), revealed impairments in sleep quality and decreases in PA levels during the lockdown in adolescent athletes using subjective tools.

Although these online surveys provide insight into the effects of home confinement on multidimensional lifestyle behaviors (***i.e.***, PA, sleep quality, dietary behaviors), the use of self-reported measurements such as the Pittsburgh Sleep Quality Index (PSQI) questionnaire and/or the self-constructed questionnaires are a primary limitation in these studies (Akbari et al., 2021[1]; Ammar et al., 2021[3]; Wingerson et al., 2021[49]). For example, previous studies showed that PSQI scores did not achieve a good correlation with polysomnography (Buysse et al., 1989[10]) or actigraphy in younger or older adults (Grandner et al., 2006[22]). In addition, the latter tool does not assess eventual napping time, which has been found to increase during the home confinement induced by COVID-19 in order to complement night sleep (Romdhani et al. 2022[37]; Smit et al., 2021[42]). Therefore, the findings of these surveys should be interpreted with caution and require complementary information through objective measurement. Smart technology provides a simple, objective, and valid tool for monitoring various health parameters (Bunn et al., 2018[9]) and may provide the best solution for obtaining objective scientific data during lockdown restrictions (Ammar et al., 2021[6]). The use of wearable technology has steadily increased and been broadly adopted since 2016, as determined by health and fitness professionals throughout the world (Thompson, 2019[45]).

The present study aimed to investigate the changes in selected PA (*i.e*., PA, step count), physiological (*i.e*., heart rate (HR), respiratory rate (RR), calories) and sleep (*i.e*.; sleep time, sleep latency, rapid eye movements (REMS) time) parameters during four consecutive months (*i.e.*, pre-confinement, 1^st^ and 2^nd^ months of confinement, and post-confinement, year 2020) in highly trained fitness coaches based on their objective smartwatch data.

## Population and Methods

### Study design and participants

This was a retrospective study analyzing data from a three-month period (23/02/2020 to 23/05/2020). The study was designed according to the guidelines of the Helsinki Declaration for conducting human experimentation and was approved by the Farhat HACHED ethical committee, Sousse, Tunisia (FH/16102020). Each participant was informed of the purpose, procedure and study details. Prior to the lockdown, participants were all continuously practicing regular PA, with an average of 12 h/week for more than the past 10 years.

### Sample size

The sample size was estimated using the following formula (Buysse et al., 1989[10]): N = [(Z_α_)^2^ × P × (1 - P) × D]/E^2^; where «*E*» was the margin of error, «*Z_α_*» was the normal deviate for one-tailed alternative hypothesis at a level of significance, «*D*» was the design (= 1 for simple random sampling), and «*P*» was the proportion of the main event of interest (*i.e.*, percentage of decrease in PA pre- and during- the home confinement). Given the pioneer character of our study, the proportion of the main event of interest was derived from a previous Spanish study (Sañudo et al., 2020[41]) aiming to evaluate the impact of COVID-19 home confinement on PA, sedentary behavior, smartphone use, and sleep patterns (n=20 young adults). In that study, the number of steps/day (objectively measured PA) significantly decreased by 68 % (p=0.68) during home confinement [from 8525±3597 (pre-lockdown) to 2754±1724 (during-lockdown)] (Sañudo et al., 2020[41]). Assuming a confidence interval of 95 % (Z_α_ = 1.64) and an «*E*» of 0.20, the total required sample size was 15 participants.

### Design

At the beginning of the study, age (year), height (cm) and weight (kg) were determined, and body mass index (kg/m^2^) was calculated. PA, physiological, and sleep data were collected over the following four consecutive months (Table 1(Tab. 1)):

- ***Month 1***: pre-confinement (up-to 23/02/2020-22/03/2020),
- ***Month 2***: first month of confinement (23/03/2020-22/04/2020),
- ***Month 3***: second month of confinement (23/04/2020-22/05/2020),
- ***Month 4***: post-confinement (23/05/2020-23/06/2020).

Ramadan month occurred during the 2^nd^ month of confinement (from 23/04/2020 to 22/05/2020). 

Data were collected using a smartwatch (Garmin Fenix 6 pro, USA). This wearable technology device is useful, reliable and valid in the assessment of sleep and PA levels (Saganowski et al., 2020[39]). Participants continuously tracked their physical and physiological activities for four consecutive months. The watch was only removed for charging purposes (~90 to 120 minutes/week). No missing data was reported other than during the charging time. Smartwatch data were downloaded using the software package (Fenix 6 Pro/6 Pro Solar software version 13.10) and were exported to a customized Microsoft Excel spreadsheet. Several investigations have also demonstrated high validity and reliability evidences of smart watch including the Garmin Fenix 6 pro or earlier models in accessing PA, step count HR, RR, calories (Navalta et al., 2020[33]) and sleep parameters (Chinoy et al., 2021[11]). A detailed description of PA (*i.e*.; PA, step count), physiological (*i.e*.; HR, RR, calories) and sleep (*i.e*.; sleep time, sleep latency, REMS time) parameters selected to be analyzed in the current study, is presented in Table 2(Tab. 2). HR was expressed in bpm (Souissi et al., 2021[44]), and as a percentage of the predicted maximal HR (PMHR (bpm) =220 - age (year)) (Robergs and Landwehr, 2002[36]).

### Statistical analyses

The normality of the data was verified by the Kolmogorov-Smirnov test. All data were expressed as means ± standard deviation (SD). One-way analysis of variance (ANOVA) with repeated measures was used to explore differences between the four consecutive months in the averaged data of PA, physiological and sleep pattern. Effect sizes were calculated as partial eta-squared (ղp^2^) to estimate the meaningfulness of significant findings. Tukey's post-hoc test was used to verify pairwise comparisons. Statistical significance was set at p<0.05. All statistical analyses were conducted using the statistical package for the social sciences (SPSS, Version 18.0, SPSS Inc., Chicago, IL, USA).

## Results

Sixteen elite fitness coaches (age: 29±3 years; height: 183±6 cm; body mass: 82±5 kg, body mass index: 24.7±1.8 kg/m^2^) participated in this retrospective study. All were Muslim and fasted during the second month of confinement (month of Ramadan: RIF).

**Figure 1:**
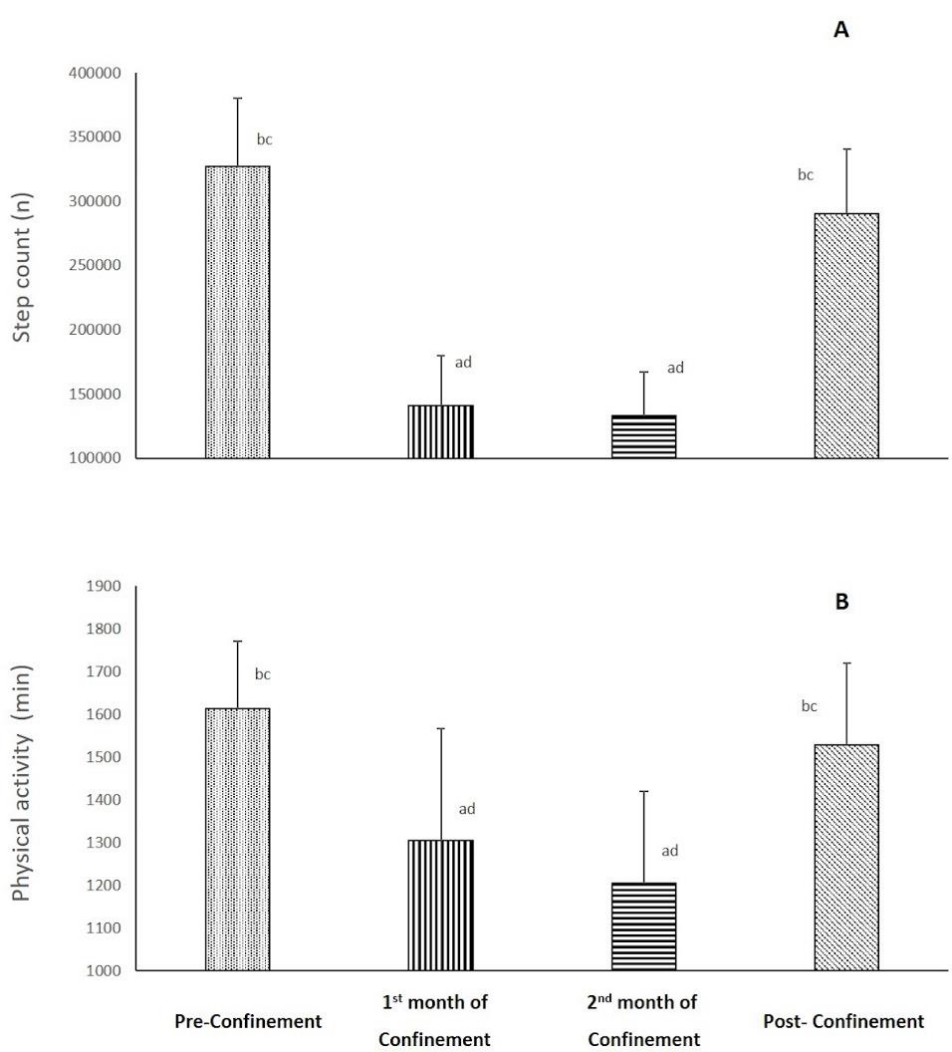
Changes in step count (A) and physical activity (B) parameters during the four consecutive months of pre-confinement, confinement and post-confinement. ^a^ Significant difference in comparison with pre-confinement (P < 0.01); ^b^ Significant difference in comparison with the 1^st^ month of confinement (P < 0.05); ^c^ Significant difference in comparison with the 2^nd^ month of confinement (P < 0.01); ^d^ Significant difference in comparison with post-confinement (P < 0.05)

PA data during the four consecutive months is presented in Figure 1(Fig. 1). There was a significant effect of consecutive months on step count and duration of PA (p< 0.001, ղp^2^= 0.98-0.83). Step count and duration of PA decreased during the 1^st^ month of confinement by 56 % and 19 %, respectively (p<0.001), and was maintained lower throughout the 2^nd^ month of confinement (Ramadan) vs. pre-confinement. During post-confinement, values of both parameters significantly increased (p<0.001, %change = +117 % and +26 %, respectively) compared to the 2^nd^ month of confinement to regain pre-confinement values.

**Figure 2:**
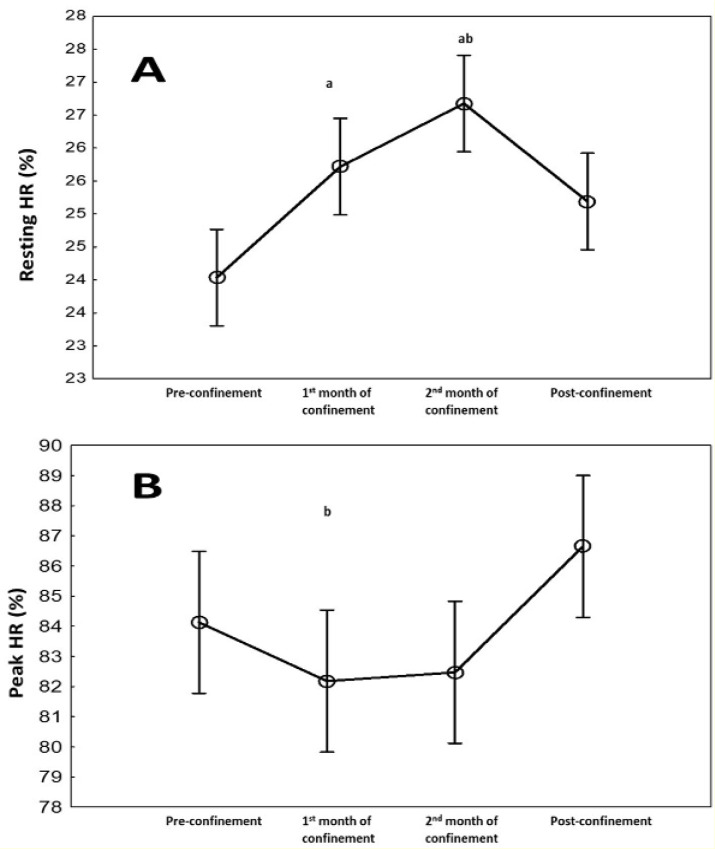
Changes in resting heart rate (bpm) (A) and peak HR (bpm) (B) during the four consecutive months of pre-confinement, confinement and post-confinement. HR: heart rate. ^a^ Significant difference in comparison with pre-confinement (P < 0.01); ^b^ Significant difference in comparison with the 1^st^ month of confinement (P < 0.05);

HR (bpm) and spent calories parameters during the four consecutive months are presented in Figure 2(Fig. 2). There was a significant effect of consecutive months on resting (p<0.001) and peak (p=0.03) HRs, and calories/day (p<0.001) data with ղp^2^=0.78-0.87. Resting HR increased (7 %, p=0.005), while calories/day decreased (-11 %, p<0.001) during the 1^st^ month of confinement vs. pre-confinement. These differences were further exacerbated during the 2^nd^ month of confinement (p<0.001 vs. pre-confinement). During post-confinement, resting HR decreased (5 %, p<0.05), and calories/day increased (20 %, P<0.001) compared to the 2^nd^ month of confinement to reach a similar level as pre-confinement. For HR peak, a significant difference was registered between the 1^st^ month of confinement and post-confinement with lower values during the confinement (p<0.05). The result indicated also that HR peak was 3 % (5 bpm) higher (p˃0.05) in the post-confinement (174±8 bpm) vs. pre-confinement (169±11 bpm).

HR (%PMHR) parameters during the four consecutive months are presented in Figure 2(Fig. 2). There was a significant effect of consecutive months on resting (p<0.05) and peak (p=0.03) HRs. Resting HR increased during the 1^st^ and 2^nd^ months of confinement vs. pre-confinement. During post-confinement, resting HR decreased compared to the 2^nd^ month of confinement to reach a similar level as pre-confinement. For HR peak, a significant difference was registered between the 1^st^ month of confinement and post-confinement with lower values during the confinement. 

**Figure 3:**
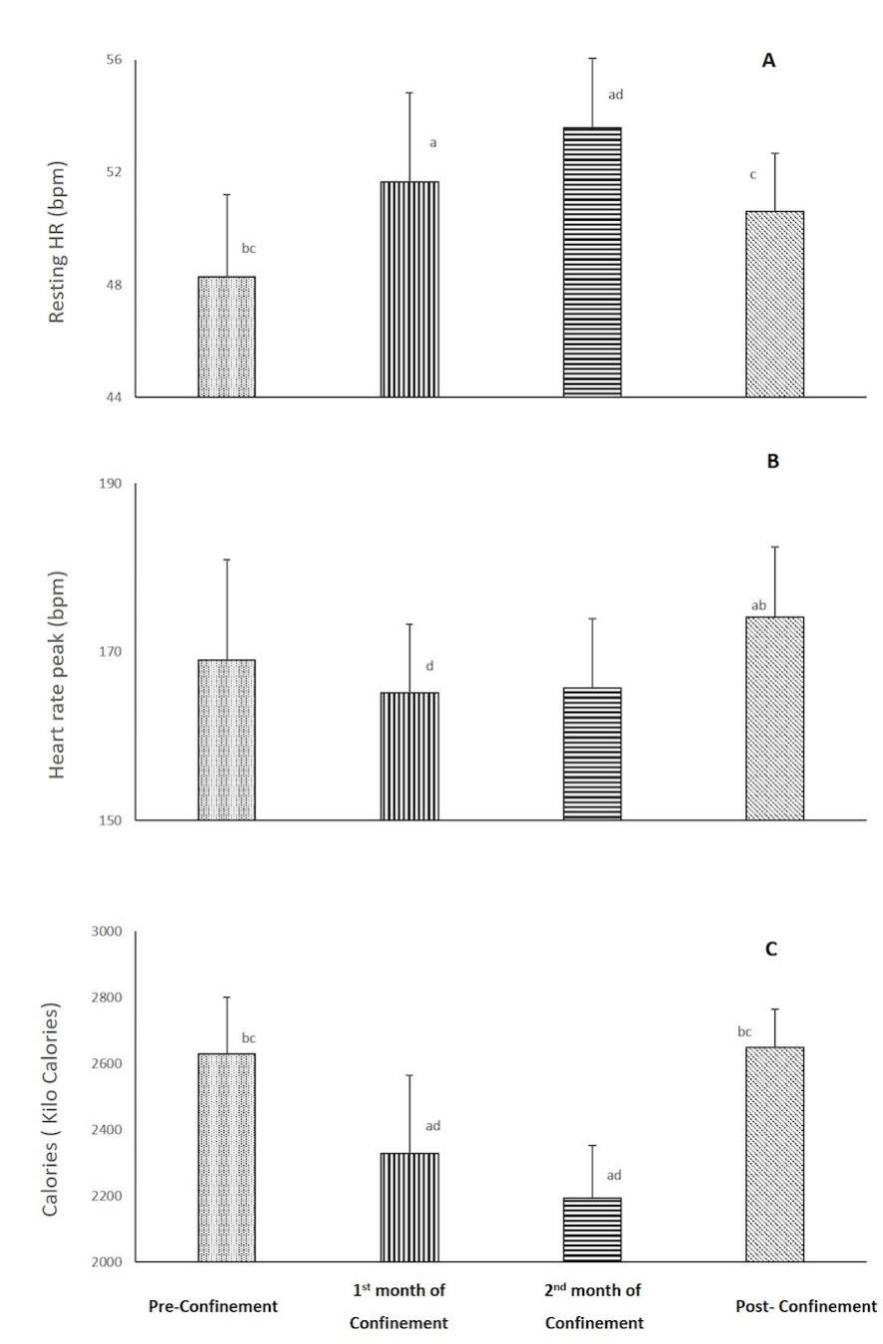
Changes in lowest (A), highest (B) and average (C) respiratory rates during the four consecutive months of pre-confinement, confinement and post-confinement. ^a^ Significant difference in comparison with pre-confinement (P < 0.01); ^b^ Significant difference in comparison with the 1^st^ month of confinement (P < 0.01); ^c^ Significant difference in comparison with the 2^nd^ month of confinement (P < 0.01); ^d^ Significant difference in comparison with post-confinement (P < 0.01)

Figure 3(Fig. 3) illustrates the RR parameters during the four consecutive months. There was a significant effect of consecutive months on the highest and average RRs (p<0.001, ղp^2^=0.62-0.71), but not on the lowest RR. Compared to pre-confinement, the highest and average RRs decreased significantly during the 2^nd^ month of confinement (p<0.001, -14 %, and -10 %, respectively). During post-confinement, values of both parameters significantly increased (p<0.001, 27 %, and 17 %, respectively) compared to the 2^nd^ month of confinement. The highest RR was 8.8 % higher (p˃0.05) in the post-confinement (22 ± 2) than in the pre-confinement (20 ± 3).

**Figure 4:**
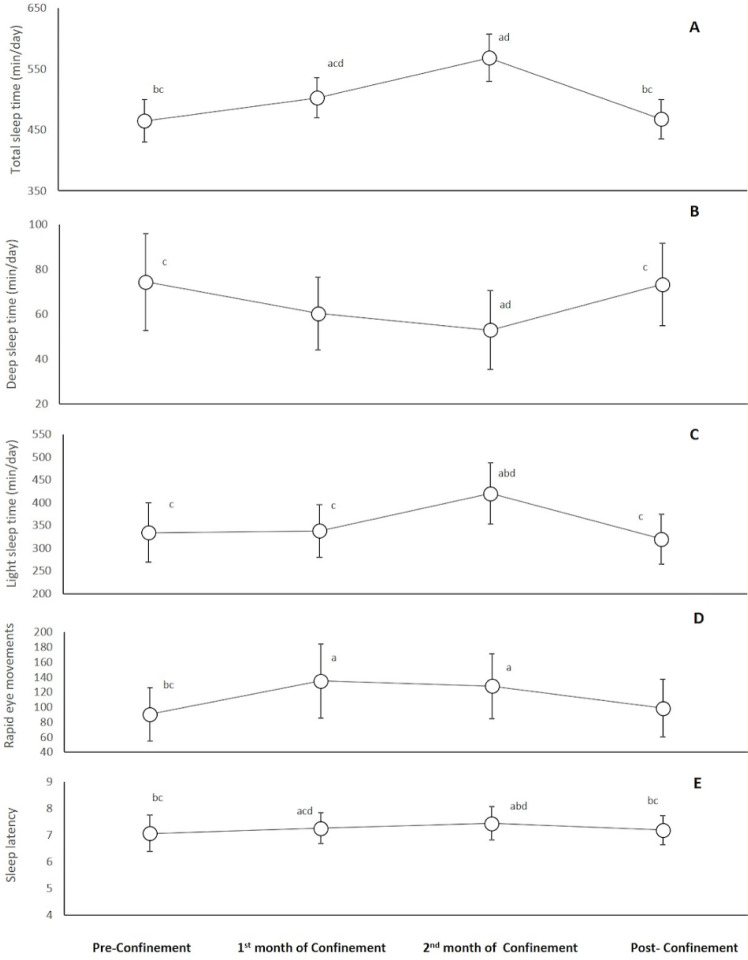
Changes in total sleep time (A), deep sleep time (B), light sleep time (C), rapid eye movements time (D), and sleep latency (E) during the four consecutive months of pre-confinement, confinement and post-confinement. ^a^ Significant difference in comparison with pre-confinement (P < 0.05); ^b^ Significant difference in comparison with the 1^st^ month of confinement (P < 0.05); ^c^ Significant difference in comparison with the 2^nd^ month of confinement (P < 0.05); ^d^ Significant difference in comparison with post-confinement (P < 0.05)

Figure 4(Fig. 4) reports a significant increase during the 1^st^ month of confinement compared to pre-confinement for total sleep (8 %, p=0.02), REMS (56 %, p=0.02) and sleep latency (523 %, p<0.001) times. These increases were further enlarged during the 2^nd^ month of confinement (p<0.001 compared to pre-confinement). For deep and light sleep times, a significant difference compared to pre-confinement was registered only during the 2^nd^ month of confinement (p=0.02 and p<0.001, respectively) with lower values of deep sleep (36 %) and higher values of light sleep (26 %) durations during the 2^nd^ month of confinement. During the post-confinement month, all sleep parameters returned to pre-confinement levels.

## Discussion

Data measured by wearable technology revealed that home confinement-induced detraining was accompanied by detrimental cardiorespiratory and sleep responses, which were enlarged during the 2^nd^ month of confinement (corresponding to RIF). However, the increased level of PA during the post-confinement month successfully reversed these negative adaptations. 

### Effect of home confinement on PA, physiological, and sleep variables 

Our results demonstrated a marked decrease in step count during the 1^st^ and the 2^nd^ month of confinement. Additionally, a decrease in PA time was observed from pre- to during-confinement. These results were expected, as all participants were banned or forbidden to leave their homes, thus preventing them from engaging in their regular PA. Moreover, the decrease in step count was more significant during the 2^nd^ month of confinement, likely due to participants training less during the lockdown. Changes in routine, increased stress, and increased anxiety are factors that decrease engagement in PA (Andreato et al., 2020[7]). Furthermore, our results reported calories spent/day decreased during the 1^st^ month of confinement and continued to decrease during the 2^nd^ month of confinement. It should be acknowledged that RIF month (*i.e.*, 4^th^ pillar of Islam, during which Muslim adults are not allowed to eat and drink from dawn to sunset during a consecutive 29 or 30 days) occurred during the 2^nd^ month of confinement. Lessan et al. (2018[27]) reported that RIF results in profound disruptions of daily activity patterns, marked by a decrease in the total number of steps per day. It appears that RIF could have exacerbated the decrease of the total number of steps during the 2^nd^ month of confinement.

The marked decrement in PA, step counts, and calories/day in response to the increase of detraining period during home confinement may also reduce cardiovascular fitness. The reduced PA reached levels well below the daily recommendation of 7500-10,000 steps per day and may further exacerbate inactivity-related health problems (Booth et al., 2017[8]). In the same context, our findings show resting HR increased during the 1^st^ and the 2^nd^ months of confinement. Narici et al. (2021[32]), reported that the spent sitting time has been linked with a reduced cardiorespiratory and cardiovascular performance. Moreover, the reduction of PA to <5000 steps/day for only a few days can impair nitric oxide mediated vasodilation (Porcelli et al., 2020[34]). Additionally, the present results showed a decrease in the average and highest RR during home confinement. The decrease in average and highest RR could be explained by the reduction in PA time and step counts and/or exercise intensity accompanied by increased behavioral stress. 

Low-intensity exercise training was recommended to prevent COVID-19 (Ghram et al., 2021[21]; Jiménez-Pavón et al., 2020[25]). Acute responses to higher intensities and volumes of exercise can involve a greater risk of illness and impaired immune function (Moreira et al., 2009[28]). In this context, Toresdahl and Asif (2020[46]) advised athletes to follow a conservative approach, limiting training sessions to <60 minutes and to <80 % of maximum ability during this time to prevent contracting COVID-19.

Our results showed a modification of sleep parameters during home confinement. Total sleep time increased by 8 % during the 1^st^ month of confinement. In addition, total sleep time increased further during the 2^nd^ month of confinement (*i.e.*, Ramadan month). The previous finding was unexpected as the results of previous meta-analyses (Faris et al., 2020[20]; Trabelsi et al., 2020[48]) concluded a decrease in total sleep time in athletes during RIF. The decrease in training load/PA levels could have blinded the effect of RIF on total sleep time. Additionally, sleep latency, and REMS increased during home confinement. The increase in the total sleep time, sleep latency, and REMS during home confinement may be due to people staying home and not waking up to go to work. However, deep sleep time decreased during home confinement, despite the increase of total sleep time. The decrease of deep sleep time is associated with an increase in sleep latency, which was not previously observed during RIF in physically active individuals (Hsouna et al., 2019[24]), indicating an impairment of sleep quality during home confinement (Akerstedt et al., 1994[2]). Again, changes in routine and increased stress and anxiety may decrease the quality of sleep and impede engagement in PA.

### Effect of post-confinement on PA, physiological, and sleep variables

Interestingly, the results showed all sleep parameters were restored during post-confinement. Furthermore, we observed an increase in PA time associated with a marked increase in step count during the 1^st^ month of post-confinement. PA time and step count increased during post-confinement reaching the same values as pre-confinement. Calories/day was also restored during home confinement; however, unlike parameters of sleep and PA, physiological parameters (Resting HR, HRpeak and highest RR) were not fully restored. Our finding highlighted higher values of HRpeak and highest RR during post-confinement vs. pre-confinement. This may be related to the adaptation difficulties of HR response with the same training load performed during post-confinement compared to pre-confinement. We hypothesized that fitness coaches had a decline in physical fitness during post-confinement due to significantly reduced training. Although they succeeded to enhance their quality of sleep and their daily PA, those parameters were still lower compared to the pre-confinement; participants also become more fatigable during post-confinement vs. pre-confinement. Our findings confirm a modified lifestyle due to home confinement can lead to serious deficits in the quality and quantity of training (Andreato et al., 2020[7]). Our work is in agreement with Narici et al. (2021[32]), who reported that living in home-confinement for several weeks represents a physiological challenge with subsequent significant health risks. It has been showed that in response to 20-day bed confinement, young healthy participants lose on average 11 % of heart volume and 28 % of maximum oxygen uptake (Saltin et al., 1968[40]). Furthermore, a period of 2-4 weeks of detraining causes a decrease in blood volume together with a decrease in haemoglobin content. These physiological changes cause a decrease in muscle capillarization and a loss of efficiency of the body temperature regulation mechanisms (Costill et al., 1985[14]; Souissi et al., 2022[43]). Furthermore, after a detraining period longer than one month, a decrease in skeletal muscle oxidative enzyme activity is observed (Costill et al., 1985[14]). This important mismatch of the aerobic system, both from the central and the peripheral perspectives, requires attention during the retraining phase.

Based on these profound physiological changes of the aerobic system, the training period during home confinement should be based on a careful choice of intensity volume, and type of training. Herrera-Valenzuela et al. (2020[23]) recommended high-intensity interval training at home to maintain cardiorespiratory endurance and physical fitness. Similarly, Jukic et al. (2020[26]) warned that detraining is one of the biggest negative consequences of the current “stay at home” confinement. Therefore, the authors recommended that the athlete's house should be equipped with cardiovascular and resistance training equipment (*e.g.*, portable cycle or rowing ergometer). It is also recommended that athletes incorporate some type of endurance exercise into their daily routine to avoid the effects of detraining during the forced quarantine. However, to avoid increased chances for upper respiratory tract infections, the athlete should train at relatively high intensities, but should not fully exhaust themselves during a training session (Moreira et al., 2009[28]; Puta et al., 2017[35]). This study also provided insight regarding the use of smart wearable devices; digital technology has become popular in the general population and has already been adopted for health monitoring. To date, smart devices have been generally used to investigate PA in daily life, showing to be effective and applicable for a large part of the healthy population and patients. Here we recommend the use of smartwatch technologies by patients, athletes, and scientific researchers based on the amount of data that they can provide and cost effectiveness. The authors, therefore, hope that, like seasonal influenza, COVID-19 will become endemic in the human population, and that COVID-19 vaccines will be incorporated as an add-on to seasonal influenza vaccinations, provided every winter for at least the next few decades (Dergaa et al., 2021[15]; Musa et al., 2021[29]). With proper immunization, the globe will return to pre-COVID levels of normalcy, and with the hope that COVID-19 lockdown scenario will not recur. 

The present study has some limitations that should be acknowledged. Our findings pertain only to physically active males. Future studies on female individuals are warranted. In addition, a control non-Muslim group (not fasting during RIF) is lacking; however, the recruitment of such group is difficult in Muslim countries.

## Perspective

We observed significant issues related to the training of fitness coaches during the COVID-19 pandemic. Fitness levels deteriorated with the confinement, even while attempting to maintain regular training. Consequently, fitness coaches significantly reduced training and experienced a decline in physical fitness and their sleep quality during home confinement. Although they enhanced their quality of sleep, daily PA, and physical fitness during post-confinement, they failed to reach the same level of performance as pre-confinement. Home-based exercise coupled with digital technology-based monitoring of physical fitness and sleep pattern could be recommended for fitness coaches and athletes during home confinement to prevent the decline in physical fitness and sleep quality.

## Notes

Ismail Dergaa and Achraf Ammar contributed equally as first author.

Amine Souissi, Mohamed Saifeddin Fessi and Khaled Trabelsi contributed equally as second author.

Helmi Ben Saad and Karim Chamari contributed equally as last author.

## Declaration

### Acknowledgments

The authors are particularly grateful to all the participants for their enthusiasm and their performance. 

### Conflict of interest

The authors declare that the research was conducted in the absence of any commercial or financial relationships that could be construed as a potential conflict of interest.

### Data availability statement

The data that support the findings of this study are openly available upon request from the corresponding author.

### Funding

This research received no specific grant from any funding agency in the public, commercial or not-for-profit sectors. 

## Figures and Tables

**Table 1:**
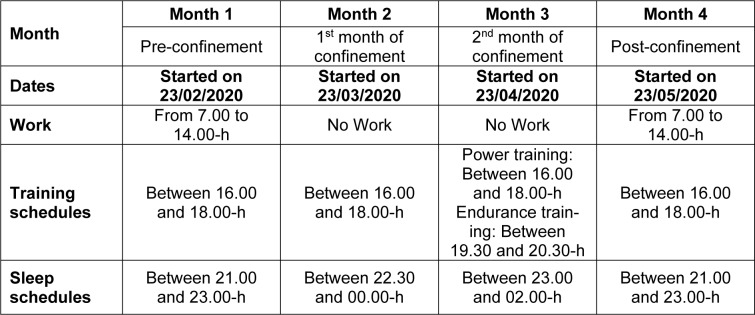
Study design

**Table 2:**
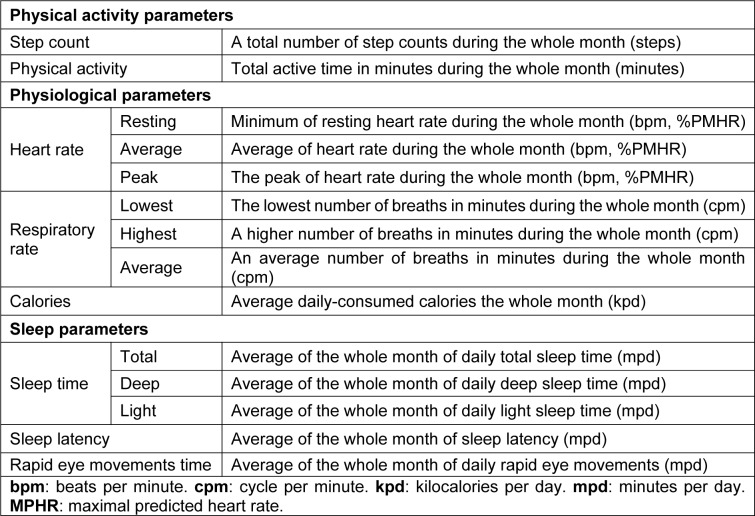
Physical activity, physiological and sleep data selected in the current study
